# Scenario-based functional modularization framework for consumer electronics using MFD and AHP: A case study on audio products

**DOI:** 10.1371/journal.pone.0339598

**Published:** 2025-12-31

**Authors:** Zhengda Wu, Jie Zhang

**Affiliations:** School of Art and Design, Beijing Forestry University, Beijing, China; West Pomeranian University of Technology, POLAND

## Abstract

Consumer Electronics (CE) currently face dual pressures: the growing demand for personalization and multi-scenario usage, and the need to enhance product sustainability. Traditional single-function designs struggle to adapt to these changes, leading to resource underutilization and the generation of significant electronic waste (e-waste). Consequently, there is an urgent need for a design method capable of systematically partitioning product functions based on distinct scenarios. However, existing modularization approaches predominantly focus on production efficiency, often failing to address multi-scenario user needs or effectively reduce e-waste. To address this gap, this paper proposes a scenario-based functional modularization framework. This framework integrates Modular Function Deployment (MFD) with the Analytic Hierarchy Process (AHP) to quantify scenario-specific requirements and establishes a Modular Scenario Relevance Matrix (MSRM) to achieve precise mapping between technical solutions and specific scenarios. A case study on audio products verified the feasibility of the framework, successfully identifying a common module and several scenario-specific functional modules (e.g., home entertainment, smart home, outdoor). This study provides designers with a systematic method for developing flexible and adaptable CE products, demonstrating how scenario-driven modularity can enhance product sustainability by reducing functional redundancy and e-waste while satisfying diverse consumer demands.

## Introduction

In the rapidly evolving Consumer Electronics (CE) market, user demands are shifting from a focus on singular functions to diverse requirements emphasizing cross-scenario experiences. This trend is driven by factors such as lifestyle transformations, the growth of outdoor activities, and the expansion of smart home ecosystems [1–4]. Product value is no longer determined solely by singular performance metrics but depends on adaptability across different “usage scenarios” [5]. However, existing product designs still primarily revolve around single scenarios, compelling consumers to repeatedly purchase products with overlapping functions to satisfy different contexts. This not only results in resource wastage but also exacerbates unsustainable lifestyles and consumption patterns [6–9].

Historically, modular design has been regarded as a key strategy for enhancing manufacturing efficiency and reducing product complexity. Its theoretical foundation emphasizes isolating changes through module boundaries to enhance reusability and improve flexibility in production and assembly [10–19]. However, this system is established on the premise of “relatively stable product functions,” with research primarily focusing on structural optimization, cost control, and production strategy adaptation [20]. For consumer electronics that emphasize usage contexts and dynamic experiences, there is a distinct misalignment between traditional modular methods and user scenario requirements: user experience focuses on contexts, changes, and tasks, whereas traditional modularity emphasizes structural partitioning and internal dependency control; the logic of the two is not entirely consistent.

On the other hand, Product Platform Strategy emphasizes achieving product family diversity through the combination of a core platform and configurable extension modules. Its theoretical focus lies in the “trade-off between commonality and differentiation.” However, existing research predominantly targets enterprise product line extensions, with less attention paid to defining platform and module boundaries based on “scenario differences.” Therefore, for consumer electronics, systematically mapping “common platforms” to “scenario-specific modules” has become a key theoretical challenge for platform strategy in the multi-scenario era.

Concurrently, sustainable design research indicates that shortened product lifecycles, functionally redundant designs, and the repetitive purchasing of multiple devices are primary drivers of electronic waste growth [6–8]. If modular design and platform strategies can adopt “scenario-based functional reuse” as their core logic, it would help reduce repetitive manufacturing and excessive purchasing, extend product lifecycles, and enhance repairability, thereby demonstrating significant potential for sustainable innovation [21–24]. In fact, there exists an intrinsic logical chain among modular theory, user experience, product platform strategy, and sustainable innovation that has not yet been systematically integrated: User Experience → Scenario Requirement Differences → Module Boundaries determined by “Usage Scenarios” rather than “Structure” → Platform constructed around “Scenario Commonality” → Functional Reuse reducing redundancy and improving sustainability. However, this logical chain has not yet formed a systematic framework within modular theory and consumer electronics applications.

Addressing this theoretical gap, this study proposes a scenario-based functional modularization framework that organically integrates scenario-specific user needs with modular theory, product platform strategy, and sustainable innovation logic. This framework establishes “usage scenarios” as the core driver for module partitioning. It employs Analytic Hierarchy Process (AHP) to quantify scenario differences, utilizes Quality Function Deployment (QFD) to map user needs to technical properties, applies Design Property Matrix (DPM) to construct the property-technical solution relationship chain, and finally proposes the Modular Scenario Relevance Matrix (MSRM) as an innovative tool to achieve systematic matching between technical solutions and scenario requirements. The core contributions of the framework include: 1) expanding modular principles from traditional “structure/manufacturing-driven” to “scenario-function-driven”; 2) defining the platform by “common functions” and modules by “differentiated scenarios,” realizing the contextualized application of platform strategy; and 3) providing structural support for sustainable product innovation by reducing repetitive purchasing and functional redundancy.

To verify the effectiveness of this framework, audio products with significant multi-scenario usage characteristics were selected as a case study. Results indicate that this method effectively identifies “cross-scenario common modules” and “scenario-specific modules,” constructing a product platform structure that possesses scalability, reusability, and combinability. This demonstrates that scenario-based modular logic not only enhances user experience consistency but also promotes sustainable innovation in consumer electronics at the product lifecycle level.

## Literature review

The concept of modular design has been widely applied in multiple industries, including mechanical equipment products manufacturing and assembly, construction, software development, biotechnology, education, and apparel [15,25–29]. In the product manufacturing sector, the core goal of modular design is to simplify production processes, reduce costs, and improve production efficiency, thereby enhancing the profitability of businesses [30,31]. In addition, modular design can improve the interchangeability of components, enabling more suppliers to provide compatible parts for other brands, thus promoting market competition and driving product upgrades.

In terms of sustainable design, modular components exhibit significant environmental friendliness during production, use, and disposal phases. For example, at the user level, modular design enhances the ease of product repair, allowing damaged components to be replaced individually, thereby extending the product’s lifespan and reducing the generation of electronic waste [21,24]. At the same time, some modular components are made from renewable or environmentally friendly materials, and by reducing raw material consumption, they further decrease the environmental burden [32].

Currently, most studies on modular design mainly focus on product structure optimization, aiming to enhance manufacturing and assembly efficiency and achieve sustainable development in production. A significant body of this research leverages matrix-based methodologies, such as Variant Mode and Effects Analysis (VMEA), QFD, Design Structure Matrix (DSM) and Modular Function Deployment (MFD) to optimize module division, reuse, and adaptability for specific physical products. Applications include optimizing automotive suspension systems [33], injection molding machine structures [34], heavy vehicle air suspension systems [15], and enhancing flexibility in products like 3D printers and electric toothbrushes [16]. Related studies have also validated virtual DSM models to analyze how structural characteristics impact modular performance [17]. Other studies explore optimization from different strategic perspectives, such as: applying genomic models and genetic algorithms to improve product sustainability and recyclability [18]; considering the effects of personalization on the product architecture lifecycle [13]; proposing design freeze strategies to reduce design iterations [35]; or using mixed-integer models to balance maintenance with design costs in modular systems [36]. While these studies effectively address the optimization of the manufacturing stage, they share a common limitation: a primary focus on production efficiency and structural optimization, with less emphasis on enhancing user needs and the product user experience.

This research focuses on CE, a field that has seen a gradual increase in variety due to continuous technological advancements and the diversification of customer requirements, covering a wide range of products from smartphones to wearable devices, and driving the sustained expansion of the market. Researchers have begun to explore how modularization can enhance product performance, reduce manufacturing costs, and improve product adaptability. For example, X Lai et al. studied the impact of modular design on the recycling strategies of discarded electronic products, noting that in a highly competitive market environment, manufacturers should improve efficiency through cooperative recycling and higher investments in modularization, while in a less competitive market, competitive recycling is more advantageous [31]. Lomax K. et al. analysed the potential of modular phones in reducing electronic waste and carbon emissions and discussed the challenges and countermeasures during the marketization process [37].

Although some brands have launched modular CE, such as Sony, Dyson, Xiaomi, and others, the application of modularization is still primarily focused on product structure optimization, with the main goal being to improve production efficiency, reduce manufacturing costs, or enhance maintainability. For example, some modular design solutions focus on dividing the internal structure of products to optimize the assembly process and improve maintainability. However, like mechanical products, the modularization study of CE generally overlooks consumer requirements, especially the adaptability issues in diverse usage scenarios. Currently, most studies focus only on designing functional modules for a single usage scenario, without fully considering the differences in consumer requirements across various environments. Therefore, how to introduce “multi-scenario adaptability” into the modular design of CE to enhance the flexibility of the product has become a direction that urgently requires further research.

The concept of “multi-scenario” has been widely applied in fields such as engineering design, urban planning, and environmental science [38–40], primarily used to assess system performance and decision optimization under different conditions. In this research, “multi-scenario” mainly refers to the functional adaptability of CE in different usage environments. As the trend of diversifying consumer demands intensifies, the adaptability and flexibility of products has become particularly important. Therefore, in the design and development of CE, modular design can be applied to enhance product adaptability in multiple environments through functional expansion modules to meet customer requirements.

Product redesign is a common strategy in product development, aimed at optimizing existing products and integrating new design concepts to achieve sustainability or circular economy goals [41,42]. For example, Teixeira et al. proposed a redesign method based on Design Thinking (DT) to help textile companies incorporate circular economy concepts into the New Product Development (NPD) process [43]; Wong et al. used a Detailed Design Model (DDM) to analyse existing product architectures, identify key geometric features, and optimize the product redesign process [44]; Cappelletti et al. proposed a method of converting composite material waste from industrial production into reusable raw materials through redesign, aiming to reduce resource waste and promote industrial symbiosis [45]. The modular design framework proposed in this research aims to perform modular redesign of existing CE, constructing a flexible modular structure to adapt to the ever-changing customer requirements. This framework not only focuses on the optimization of product structure but also emphasizes the adaptability of products in multiple usage scenarios, enhancing the functional expansion capabilities of CE and promoting sustainable production and consumption patterns.

## Methodology

The method used in this research is divided into three steps. First, customer requirements acquisition, identifying the product functions needed by customers in different usage scenarios. Second, customer requirements analysis, transforming them into the basis for modularization. Finally, modularization, where product components are divided according to different usage scenarios, and the results are analysed and optimized to better meet actual requirements.

### Requirement gathering

The purpose of this step is to acquire the product’s usage scenarios and the specific customer requirements in each scenario. The number of usage scenarios determines the scope and direction of product modularization, while the needs in each scenario provide concrete basis and reference for modularization. Methods for gathering customer requirements can be divided into two major categories: quantitative analysis and qualitative analysis. Quantitative analysis includes methods such as surveys, data mining, and market analysis [46,47], while qualitative analysis includes methods such as in-depth interviews, focus group discussions, and observation. Overall, quantitative analysis can provide general conclusions through the processing of large amounts of data and is suitable for obtaining a broad understanding of consumer requirements (CR), whereas qualitative analysis helps to deeply explore users’ psychological and behavioural motivations, providing deeper user insights.

This research uses quantitative analysis to derive CR in specific usage scenarios, with surveys as the primary data-gathering method.

### Analytic hierarchy process

To systematically quantify and rank the complex and diverse Customer Requirements (CR) across different scenarios, this study selected the AHP. AHP is a well-established tool widely applied in decision analysis, making it particularly suitable for determining the relative weights of different criteria.

AHP was first introduced by Saaty and has been widely applied in decision analysis [48]. The AHP method determines the weights of different requirements by comparing the various needs of a specific object. Many studies have shown that AHP is a well-established multi-criteria decision-making method that enables structured problem decomposition and quantitative weighting of evaluation criteria. Prior studies commonly adopt AHP to identify key factors, rank alternatives, and enhance decision transparency and reliability under conditions of uncertainty [49–51]. AHP is also frequently integrated with complementary approaches such as TOPSIS, QFD, and data-driven techniques to improve indicator extraction, requirement translation, and priority determination, thereby supporting more comprehensive decision analysis [52–54]. Overall, AHP offers clear structure, ease of implementation, and strong compatibility with other methods, making it a robust tool for addressing complex multi-criteria decisions. Based on this characteristic, this research selects AHP as the first step in CR analysis, providing more valuable input for the subsequent QFD in the Design Property Matrix (DPM) regarding CR gathering and analysis.

Specifically, this research utilizes AHP to analyse the CR data collected from the survey, calculating the weights and ranking the CR across different scenarios. This process identifies the most important requirements in each scenario, which are then used to inform the modular design. The final weight values of these requirements are directly applied in the subsequent QFD and DPM analyses to ensure that the modular design precisely aligns with the CR.

### Modular function deployment

MFD, as a mature and well-established modular research method, is a systematic design process that typically includes steps such as requirement identification, functional decomposition, and module design. In this process, tools such as QFD, DPM, and MIM are integrated to gradually transform CR into driving factors for modular design.

Many studies have shown that MFD, as a systematic design process, is both flexible and extensible. On one hand, MFD is often integrated with other analytical tools to solve specific engineering or decision-making problems. Examples include combining it with the DSM to optimize the modularization of physical systems [15], with the Product Line Commonality Index (PCI) to optimize costs via component commonality [55], or with the Fuzzy Analytic Hierarchy Process (FAHP) to support group decision-making in complex systems [56]. On the other hand, the MFD framework itself is frequently modified or adapted to fit different industrial contexts and strategic goals. This includes applications in non-assembly products within the process industry [57], modifications to suit brownfield manufacturing systems [58], and extensions (such as integrating R-imperatives) to address the full product lifecycle and sustainability goals [59]. Furthermore, the Modular Function Deployment Adapted (MFDA) approach was proposed to select appropriate steps and tools based on project complexity and novelty [60]. This body of research demonstrates that MFD is not a rigid process, but rather a flexible framework that can be adapted and integrated to meet diverse product development needs.

This research focuses on CE, and due to the differences in the number and complexity of internal components compared to mechanical products, and the fact that it does not involve analysis at the corporate strategy level, appropriate adjustments have been made to the MFD. Specifically, this research emphasizes the application of QFD and DPM to optimize the product modular design process.

#### Quality function deployment.

As one of the core tools in the MFD framework, QFD is used to translate CR into specific product properties (PP). QFD itself, as a classic customer-centric tool, is integrated with other methods in many studies to solve more complex challenges. Examples include combining it with TRIZ (Theory of Innovation Problem Solving) to achieve radical innovations through a two-phase House of Quality (HoQ) design [61], or integrating it with FMEA (Failure Mode and Effect Analysis) to balance customer satisfaction with risk management during product upgrades [62]. Furthermore, to address decision complexity and data uncertainty, QFD is often combined with techniques like AHP and PCA (Principal Component Analysis) to reduce matrix complexity and enhance decision-making efficiency, a practice that has been validated in the consumer electronics sector [52]. Concurrently, fuzzy approaches are also used to integrate tools like QFD, Kano, and SERVQUAL to more accurately handle the ambiguous information derived from customer perceptions [63].

In QFD, it is necessary to first identify the PP, which are the technical means by which the product meets CR. Then, PP, CR, and their weights are filled into the judgment matrix, with 1, 3, and 9 representing the levels of importance, to reflect the relationship between PP and CR.

QFD analysis provides a ranking of PP and identifies the PP that have the greatest impact on key CR. These high-scoring PP will be the key focus in the subsequent DPM, guiding designers to concentrate on these metrics and prioritize optimization during the product design process.

#### Design property matrix.

The DPM constitutes another critical step within MFD, serving to correlate PP with Technical Solutions (TS). For instance, Forti et al. [15], in their research integrating DSM with MFD, utilized DPM to integrate engineering perspectives and support modularization decisions. Similarly, Asif et al. [22] applied DPM to analyze the associations between technical solutions and product properties, aiming to identify potential module clusters in multiple-lifecycle products.

The main goal of DPM is to determine which TS can effectively meet PP, ensuring that the design is more rational while satisfying CR. The specific steps are as follows: first, obtain the TS for the product. For CE, the basic unit is a component rather than a part. Next, construct the DPM by filling in PP and TS in the matrix, and use 1, 3, and 9 to represent the levels of importance. Afterward, analyse the impact of PP by calculating the total score to determine the extent to which a particular PP is influenced by all TS. Finally, optimize the modular design by identifying TS that have little or no impact on the PP, thereby reducing unnecessary designs.

DPM is an important tool in engineering design for achieving a “customer-centered” approach, effectively guiding product design optimization, improvement, and modularization decisions.

#### Modular scenario relevance matrix.

According to the standard MFD implementation process, the process of modularization typically relies on the analysis data from MIM and DPM as the judgment condition. However, since this research does not involve analysis at the corporate strategy level, the judgment of the MIM is not applicable. To more precisely divide the modular structure that meets different usage scenarios, this research has designed the MSRM. The design of the MSRM draws upon the principles of other matrix-based analysis methods in engineering design, aiming to systematically evaluate the relevance between TS and different usage scenarios. Its main goal is to: identify common modules, which are modules that can be reused across multiple scenarios; identify specific modules, which are modules that are only applicable to specific scenarios; and optimize pending modules by combining the results of QFD and DPM analysis with the current state of the actual product.

### Case study method

**Fig 1:**
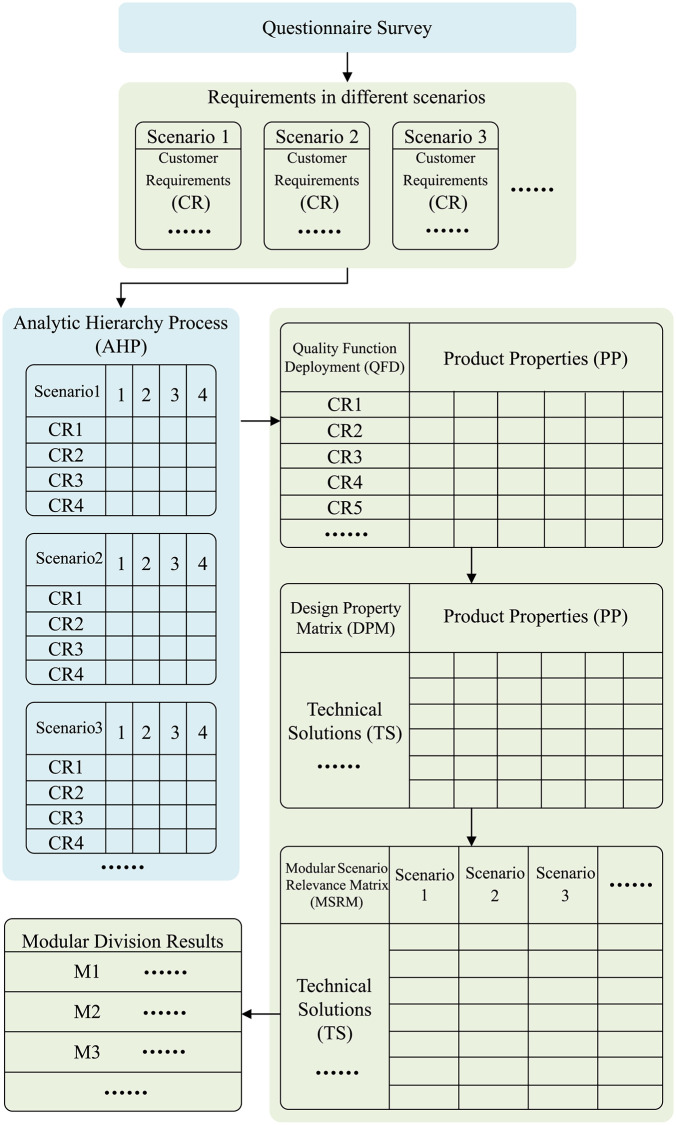
Functional modularization framework.

The case study method is widely used in product modularization-related research to verify the application of theories in practical cases [15,16,64]. In this research, an existing CE will be selected as a case study, and modular redesign will be conducted based on the framework to verify the effectiveness of the framework. To simulate the final modular design effects and test its practical application, this research will also use digital modeling techniques. The framework of this research is shown in Fig 1.

## Case study

The method in this research is applicable to functional modular CE with multi-scenario usage requirements. These products have wide application scenarios, diverse types, and sufficient internal space to accommodate multiple functional modules. For example, audio products, projectors, IoT devices and some wearable devices require flexible functional support in different usage scenarios, and therefore, they need to have enough internal space for modularization, effectively adapting to different customer requirements. In contrast, high-integration products such as smartphones, laptops, and digital cameras have very compact internal spaces and rely on high integration to ensure performance and portability. Modular design may lead to space wastage and affect performance, making it unsuitable for the method proposed in this research.

This research selects audio products as a case to explore the advantages of the proposed method in meeting the diverse customer requirements and multi-scenario applications in audio products. As consumers’ demand for functional diversity and scenario flexibility continues to grow, the application fields of audio products have gradually expanded, including home entertainment, smart homes, and outdoor activities. This makes audio products an ideal subject for studying modular design, as they effectively demonstrate the potential of modular design to enhance product flexibility and meet personalized requirements.

### AHP analysis of customer requirements

To understand consumers’ real needs, we distributed an anonymous online survey and successfully collected 108 responses. This study has received ethical exemption approval from the Ethics Committee of Beijing Forestry University. The survey was conducted anonymously between January 18 and January 26, 2025. All participants were adults and participated voluntarily. Before beginning the questionnaire, they were provided with an introductory statement explaining the purpose of the study, the voluntary nature of participation, and assurances of anonymity. No personal identifiable information was collected. Completion of the questionnaire was taken as implied informed consent.

The demographic profile of the valid respondents (N = 94) aligns closely with the primary target market for modular consumer electronics. Specifically, 85.7% of participants were aged between 18 and 35 (18–25: 31.4%; 26–35: 54.3%), representing the core consumer group for innovative electronic products. In terms of usage habits, 64.9% of users reported frequent use of audio products (34.1% daily and 30.8% several times a week). Furthermore, the participant composition was diverse, including not only potential consumers but also audio enthusiasts (audiophiles) and industry practitioners (including sales and R&D personnel). This composition ensures that the derived requirements reflect both genuine market expectations and professional technical insights, thereby securing the validity and representativeness of the AHP analysis despite the limited sample size.

Before distributing the survey, we conducted a study on existing audio products in the market and identified five common audio usage scenarios: outdoor usage scenario, smart home scenario, home entertainment scenario, daily leisure usage scenario, and work meeting scenario. To simplify the operational process, we processed the CR obtained from the survey. By analysing the functional requirements, we eliminated those with fewer needs and discarded the work meeting scenario, which had the least demand, to avoid overdesign and make the subsequent analysis process more reasonable. The results are shown in Table 1.

**Table 1:** Customer requirements and corresponding codes for each usage scenario.

Usage Scenarios	Customer Requirements Codes	Customer Requirements
Daily Leisure Usage Scenario	CR1	Easy operation
	CR2	Stylish appearance
	CR3	Long playback time
	CR4	Multiple sound effect adjustments
Home Entertainment Scenario	CR5	High-quality sound
	CR6	Surround sound experience
	CR7	Multiple connection options
	CR8	Remote and mobile control
Smart Home Scenario	CR9	Voice control
	CR10	Smart home integration
	CR11	Wireless streaming
	CR12	Multi-room synchronization
Outdoor Usage Scenario	CR13	Wireless connection
	CR14	Long usage time
	CR15	Easy to carry
	CR16	Water and dust resistance

For the four scenarios in Table 1, AHP was applied to analyse the CR for each scenario and determine the weight of each requirement. The functional requirements for each scenario were then ranked based on their weights. The consolidated results are shown in Table 2. From Table 2, the ranking of the requirements obtained after scoring is largely consistent with the order chosen by the respondents in the survey.

**Table 2:** Results of AHP analysis: Requirement weights, ranking, and consistency check for each scenario.

Usage Scenarios	Customer Requirements Codes	Weights	Customer Requirements	Consistency check
Daily Leisure Usage Scenario	CR1	0.362	Easy operation	Maximum eigenvalue: 4.10
	CR2	0.35	Stylish appearance	Consistency Index (CI): 0.032
	CR4	0.156	Multiple sound effect adjustments	Random Consistency (RI): 0.89
	CR3	0.132	Long playback time	Consistency Ratio (CR): 0.036
Home Entertainment Scenario	CR5	0.538	High-quality sound	Maximum eigenvalue: 4.05
	CR6	0.235	Surround sound experience	Consistency Index (CI): 0.017
	CR8	0.142	Remote and mobile control	Random Consistency (RI): 0.89
	CR7	0.085	Multiple connection options	Consistency Ratio (CR): 0.019
Smart Home Scenario	CR9	0.371	Voice control	Maximum eigenvalue: 4.01
	CR10	0.277	Smart home integration	Consistency Index (CI): 0.005
	CR11	0.267	Wireless streaming	Random Consistency (RI): 0.89
	CR12	0.085	Multi-room synchronization	Consistency Ratio (CR): 0.005
Outdoor Usage Scenario	CR13	0.39	Wireless connection	Maximum eigenvalue: 4.04
	CR14	0.262	Long usage time	Consistency Index (CI): 0.013
	CR15	0.244	Easy to carry	Random Consistency (RI): 0.89
	CR16	0.104	Water and dust resistance	Consistency Ratio (CR): 0.014

The evaluation data (AHP, QFD, DPM, MSRM) for this study were sourced from a panel of three experts. This panel comprised an audio industry professional, a consumer electronics sales specialist, and a senior digital product reviewer, ensuring a diversity of evaluation perspectives. All personal information of the experts was anonymized. For the AHP analysis, data were aggregated by calculating the geometric mean of the experts’ independent judgments. For the scoring in QFD, DPM, and MSRM, the final scores were determined through organized panel discussions to reach a consensus.

### QFD relates customer requirements to product properties

After analysis, CR were converted into quantifiable and controllable PP. These properties typically include technical specifications, performance requirements, and engineering characteristics such as durability, battery life, and portability, which specifically describe the features the product should have to meet CR. Therefore, in QFD, the first step is to convert CR into PP. Different CR may correspond to the same PP. Given that this research focuses on modular design based on modular units, the goal is to clarify the logic and concepts of product modularization at the early stages. As a result, PP related to circuit control and similar aspects are downplayed, while function-related PP are highlighted. Through screening and optimization, the final list of PP was obtained, as shown in Table 3.

**Table 3:** Product property and corresponding codes.

Product Properties	Product Properties Codes
High-fidelity sound quality	PP1
Sound customization	PP2
Surround sound	PP3
Voice assistant	PP4
Remote control and mobile app control	PP5
Intuitive user interface	PP6
Wireless and wired connections	PP7
Multi-room synchronization feature	PP8
Quick pairing	PP9
Long battery life	PP10
Fast charging	PP11
Modern design aesthetics	PP12
Convenience and ergonomics	PP13
Durability and sealed structure	PP14
Automated scene configuration	PP15
Multi-protocol support	PP16

**Fig 2:**
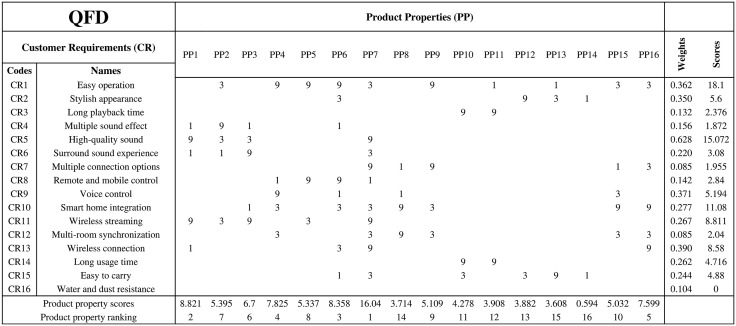
Quality function deployment (QFD).

After obtaining the PP, CR are associated with PP using a judgment matrix, and the strength of their relationships is determined through scoring, as shown in Fig 2. A score of 1 represents a weak relationship, 3 represents a moderate relationship, and 9 represents a strong relationship, with blanks indicating no relationship. For example, for CR2 (Stylish appearance), there is a strong correlation with PP12 (Modern design aesthetics), so a score of 9 is assigned. However, PP6 (Intuitive user interface) and PP13 (Convenience and ergonomics) have a weaker correlation, so a score of 3 is assigned. By placing the weights of the CR obtained from AHP on the right side of the matrix, a more accurate score for both CR and PP can be obtained. The weights of the CR are not normalized because the requirements in the four scenarios are independent and there is no interdependence. The PP scores are calculated by multiplying the scores in the matrix by the weights of the CR and then summing them. For example, the score for the first column, PP1 (High-fidelity sound quality), is 8.821= (1 × 0.156 + 9 × 0.628 + 1 × 0.22 + 9 × 0.267 + 1 × 0.390). The higher the PP score, the more it satisfies the CR and should be prioritized. The CR score is the sum of the scores in each row multiplied by the CR weight. For example, the score for CR1 (Easy operation) is 18.1 = (3 + 9 + 9 + 9 + 3 + 9 + 1 + 1 + 3 + 3) ×0.362. The higher the CR score, the better the final product design will meet CR.

As shown in Fig 2, although CR3 (Long playback time) and CR14 (Long usage time) have similar meanings, they were not merged or removed to ensure the accuracy of the CR weights. From Fig 2, the highest-scoring PP is PP7 (Wireless and wired connections), which involves multiple CR. Additionally, PP such as PP1 (High-fidelity sound quality), PP6 (Intuitive user interface), PP4 (Voice assistant), and PP16 (Multi-protocol support) are strongly correlated with CR, and therefore, these PP should be given priority in the subsequent design stages.

### DPM relates product properties to technical solutions

After completing the QFD analysis, the relationship between CR and PP has been clarified. Next, DPM is used to link the PP with the functional components of the actual product which are TS, to identify the TS that most influence the PP and focus on these solutions to maximize the satisfaction of CR. When determining TS, it is essential to ensure that all necessary solutions are included, while avoiding unnecessary redundant solutions. Therefore, the first step is to analyse the expected product forms of the audio products in the four scenarios and select representative existing products from the market for decomposition, extracting the necessary TS. This method of modular study based on existing products is called bottom-up analysis [65]. In addition to bottom-up analysis, there is also top-down analysis, where TS are identified by starting from the product functions and gradually breaking them down.

As described in QFD, this design mainly focuses on the structure and modularization, rather than delving into the circuit aspects. Therefore, circuit and chip-related components are somewhat downplayed. However, large components, such as power amplifiers and high-power transformers, are still considered. By analysing existing audio products, we selected four representative products, each corresponding to the needs of different scenarios:

Audio products for the daily leisure usage scenario: These are primarily designed for indoor home environments, emphasizing the integration of shape and interior design. They are based on home audio products, such as those from Marshall.

Audio products for the home entertainment scenario: These are primarily active three-way high-fidelity audio systems, referencing home theatre audio products available on the market, such as those from SONY.

Audio products for the smart home scenario: These are smart speakers with voice assistants, referencing smart speaker products available on the market, such as Apple’s HomePod.

Audio products for the outdoor usage scenario: These are outdoor audio systems with a certain level of dust and water resistance and heavy bass effects, referencing outdoor audio products such as those from JBL.

Based on the above analysis, the corresponding TS are shown in Table 4.

**Table 4:** Technical solutions and corresponding codes.

Technical Solutions	Technical Solutions Codes
6.5-inch driver	TS1
3-inch driver	TS2
1-inch driver	TS3
Battery	TS4
Small circuit components	TS5
Enclosure	TS6
Microphone array	TS7
Interactive display	TS8
Physical buttons	TS9
Indicator lights	TS10
Wired input port	TS11
Wireless input module	TS12
Remote control	TS13
High-power supply	TS14
Low-power supply	TS15
Bass reflex port	TS16
Independent crossover	TS17
Independent power amplifier	TS18
LED accent lights	TS19
Passive driver	TS20
Waterproof gasket	TS21
Dustproof grille	TS22
Handle	TS23

**Fig 3:**
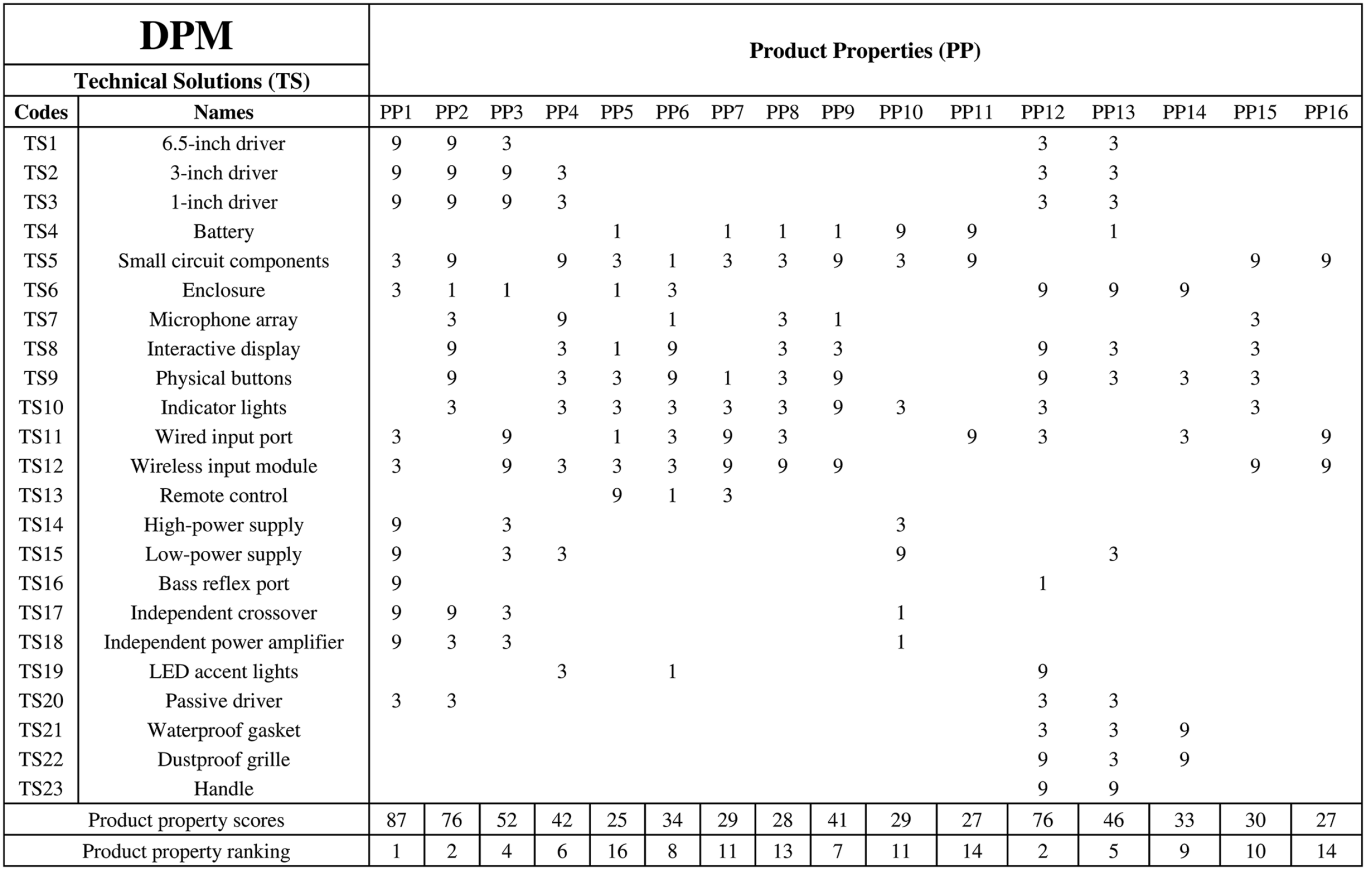
Design property matrix (DPM).

The main goal of DPM is to identify the TS that can effectively achieve PP by determining the relationships between PP and TS. This ensures rationality and efficiency of the design while meeting CR. The PP and TS are placed into the DPM, resulting in the matrix shown in Fig 3.

The scores of 1, 3, and 9 in DPM represent the correlation between TT and PP, with 1 indicating weak correlation, 3 indicating moderate correlation, and 9 indicating strong correlation. A blank indicates no relationship. The score for each PP is the sum of the scores in each column. From the scores, PP1 (High-fidelity sound quality) is associated with the most TS, which directly impact the realization of PP1. Based on the score for PP1 in QFD, to maximize the satisfaction of CR, the subsequent stages should analyse and verify whether these TS can be grouped into the same module. Additionally, the highest-scoring PP in QFD also include PP7 (Wireless and wired connections), PP6 (Intuitive user interface), PP4 (Voice assistant), and PP16 (Multi-protocol support). In the subsequent modularization stage, priority should be given to whether these TS can be combined into the same module to better meet CR.

### MSRM divides modules

To more effectively achieve modularization for different usage scenarios, this research designed a judgment matrix, the MSRM, to allocate appropriate TS for different scenarios. The main contents of the matrix include four columns:

- TS: Lists all the TS input into the DPM.
- Scenario Relevance Judgment: The relevance of each TS in different usage scenarios, with scores of 1, 3, and 9 representing weak, moderate, and strong correlations, respectively.
- TS Scores: After scoring each TS in different scenarios, the scores are summed to obtain the total score.
- Component Type Judgment: Based on the total score, determine whether the TS belongs to a common module or a specific module. Common modules apply to all scenarios, while specific modules are only applicable to a particular scenario.

**Fig 4:**
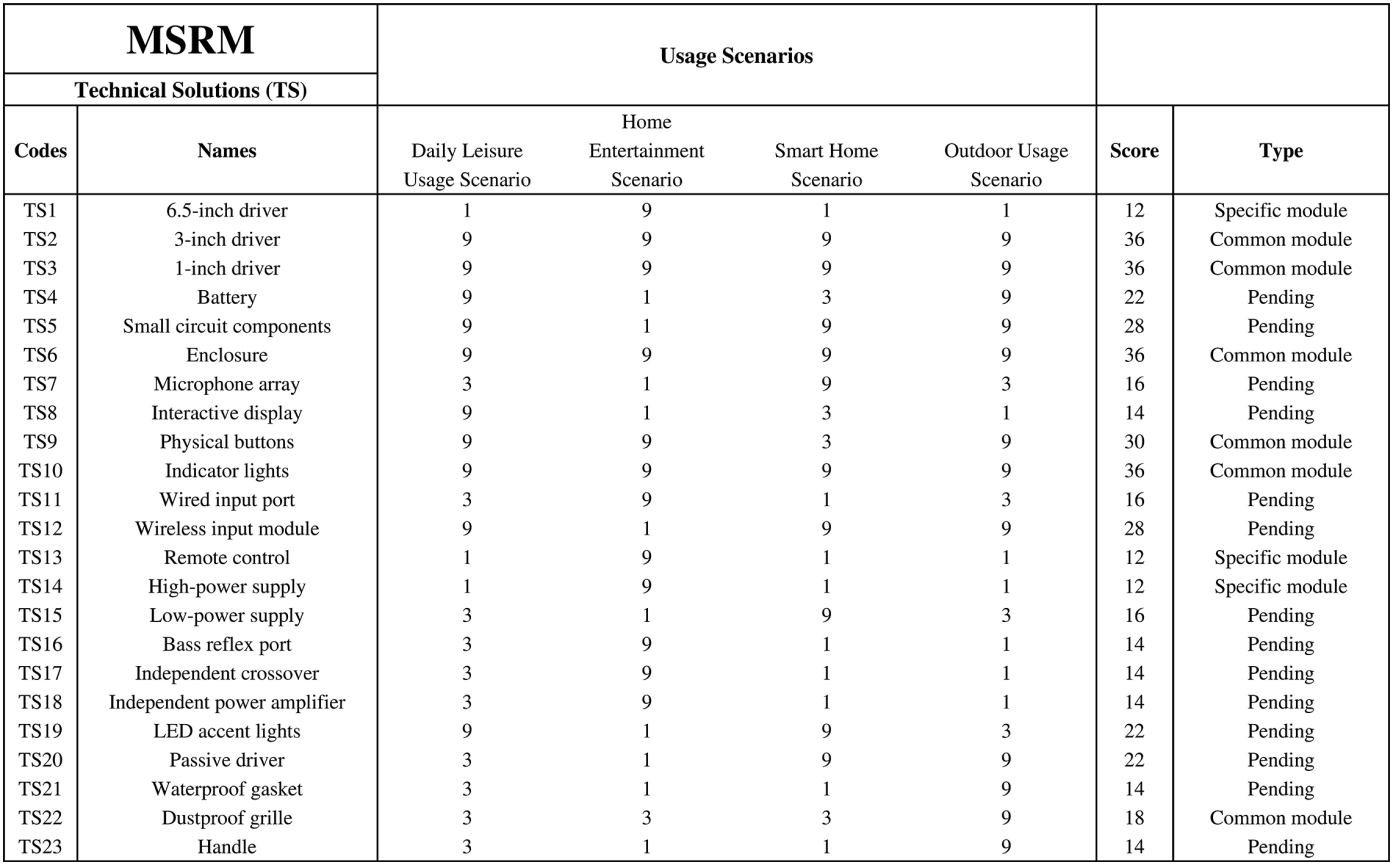
Modular scenario relevance matrix (MSRM).

For example, in the case study of this paper, scores are given for the four usage scenarios. If a TS is applicable to all scenarios, each scenario will have a score of 9, resulting in a total score of 36, which is defined as a common module. If the TS is only applicable to one scenario, the other scenarios will have a score of 1, resulting in a total score of 12, which is defined as a specific module. If the score falls between 12 and 36 and no score 1 appears which means it is related to all scenarios, it can still be considered a common module. If a score 1 appears, it indicates that some scenarios have weak relevance, and further consideration is needed to determine whether the TS is a common module. If uncertain, it should be marked as “pending.” The final MSRM is shown in Fig 4.

Through the MSRM analysis, the TS included in the common modules are as follows: 3-inch driver, 1-inch driver, enclosure, physical buttons, indicator lights, and dustproof grille. These TS can be grouped into one functional module, designated as m1, which serves as the primary module for this modular audio product and is applicable to all usage scenarios. The 6.5-inch driver is a specific module, targeted at the home entertainment scenario. Additionally, TS13 and TS14 are also specific to the home entertainment scenario. Therefore, these TS are temporarily grouped into one module, m2.

In Fig 4, there are several pending TS. These TS will be analysed one by one to achieve the modularization process. TS4 (Battery) is a necessary component for both the daily leisure usage scenario and the outdoor usage scenario. Therefore, this component should be bound to the audio product forms in these two scenarios, meaning it can either be treated as a standalone battery module supporting these two scenarios or combined with module m1 as a whole. For now, the battery is considered module m3. TS5 (Small circuit components), TS12 (Wireless input module), and TS20 (Passive driver) all received scores of 3 or 9 in all scenarios except for the home entertainment scenario, so they can be grouped into module m4. TS11 (Wired input port), TS16 (Bass reflex port), TS17 (Independent crossover), and TS18 (Independent power amplifier) all received a score of 9 in the home entertainment scenario, with low relevance in other scenarios, so they can be treated as module m5. TS7 (Microphone array) and TS15 (s Low-power supply) both scored 9 in the smart home scenario, but low scores in other scenarios, so they are grouped into module m6. TS8 (Interactive display) and TS19 (LED accent lights) are both related to the daily leisure and smart home scenarios, so they are temporarily assigned as module m7. Finally, TS21 (Waterproof gasket) and TS23 (Handle) are assigned as modules m8 and m9, respectively. The preliminary analysis results are shown in Table 5.

**Table 5:** Preliminary modularization results.

Module Codes	Technical Solutions Codes	Technical Solutions (TS)
m1	TS2	3-inch driver
	TS3	1-inch driver
	TS6	Enclosure
	TS9	Physical buttons
	TS10	Indicator lights
	TS22	Dustproof grille
m2	TS1	6.5-inch driver
	TS13	Remote control
	TS14	High-power supply
m3	TS4	Battery
m4	TS5	Small circuit components
	TS12	Wireless input module
	TS20	Passive driver
m5	TS11	Wired input port
	TS16	Bass reflex port
	TS17	Independent crossover
	TS18	Independent power amplifier
m6	TS7	Microphone array
	TS15	Low-power supply
m7	TS8	Interactive display
	TS19	LED accent lights
m8	TS21	Waterproof gasket
m9	TS23	Handle

Through preliminary analysis, a general concept for the modularization process has been defined. Based on existing products in the market and the priority of TS related to CR obtained earlier, the modularization is further optimized. This primarily involves evaluating whether the modularization is reasonable and whether different modules can be combined into a single module to reduce the complexity for consumers during use, as well as the cost during production.

For m1, it includes TS2 and TS3, which are strongly related to PP1, PP2, and PP3, and strongly correlated with CR. This means that the handling of TS2 and TS3 has a significant impact on CR. Therefore, in m1, TS2 and TS3 are the core elements, and other TS should be configured around these two components.

For m4, since it occupies little space and its usage scenarios are more mobile, combining the circuit and speaker as a whole is more advantageous for cost control. Therefore, it is combined with m1 to form a new module, M1.

For m7, TS8 and TS19 are more frequently used in indoor scenarios, adding to the fun of usage. Thus, it is defined as M2. When combined with M1, it can meet the requirements of the daily leisure usage scenario.

For m2, after analysing existing products, it can be combined with m5 to form a high-fidelity audio module, M3. When combined with M1, which includes TS2 and TS3, it can achieve complete functionality.

For m6, since TS7 and TS15 are strongly associated with the smart home scenario, it is defined as M4. When switching to the smart home scenario, it can be combined with M1 to meet the requirements of a smart speaker.

For m8, if it were treated as a standalone module, it would increase usage complexity, and since this module is specific to the outdoor usage scenario, it is incorporated into M1. For m9, it can be treated as a standalone module, M5, allowing users to choose according to their usage needs.

For m3, if it was a standalone module, when the user switches to the daily leisure usage scenario or the outdoor usage scenario, it would need to combine with M1. Considering that this module mainly affects PP10 and PP11, and these two PP have relatively lower weights in terms of CR, in order to reduce production costs and enhance user convenience, m3 is integrated as a component into M1.

Additionally, TS6 (Enclosure) is a relatively unique TS, as it is required in every module except M5. Therefore, TS6 will appear in every module. The optimized modularization results are shown in Table 6.

**Table 6:** Final modularization results.

Module Codes	Technical Solutions Codes	Technical Solutions (TS)
M1	TS2	3-inch driver
	TS3	1-inch driver
	TS4	Battery
	TS5	Small circuit components
	TS6	Enclosure
	TS9	Physical buttons
	TS10	Indicator lights
	TS12	Wireless input module
	TS20	Passive driver
	TS21	Waterproof gasket
	TS22	Dustproof grille
M2	TS6	Enclosure
	TS8	Interactive display
	TS19	LED accent lights
M3	TS1	6.5-inch driver
	TS6	Enclosure
	TS11	Wired input port
	TS13	Remote control
	TS14	High-power supply
	TS16	Bass reflex port
	TS17	Independent crossover
	TS18	Independent power amplifier
M4	TS6	Enclosure
	TS7	Microphone array
	TS15	Low-power supply
M5	TS23	Handle

**Fig 5:**
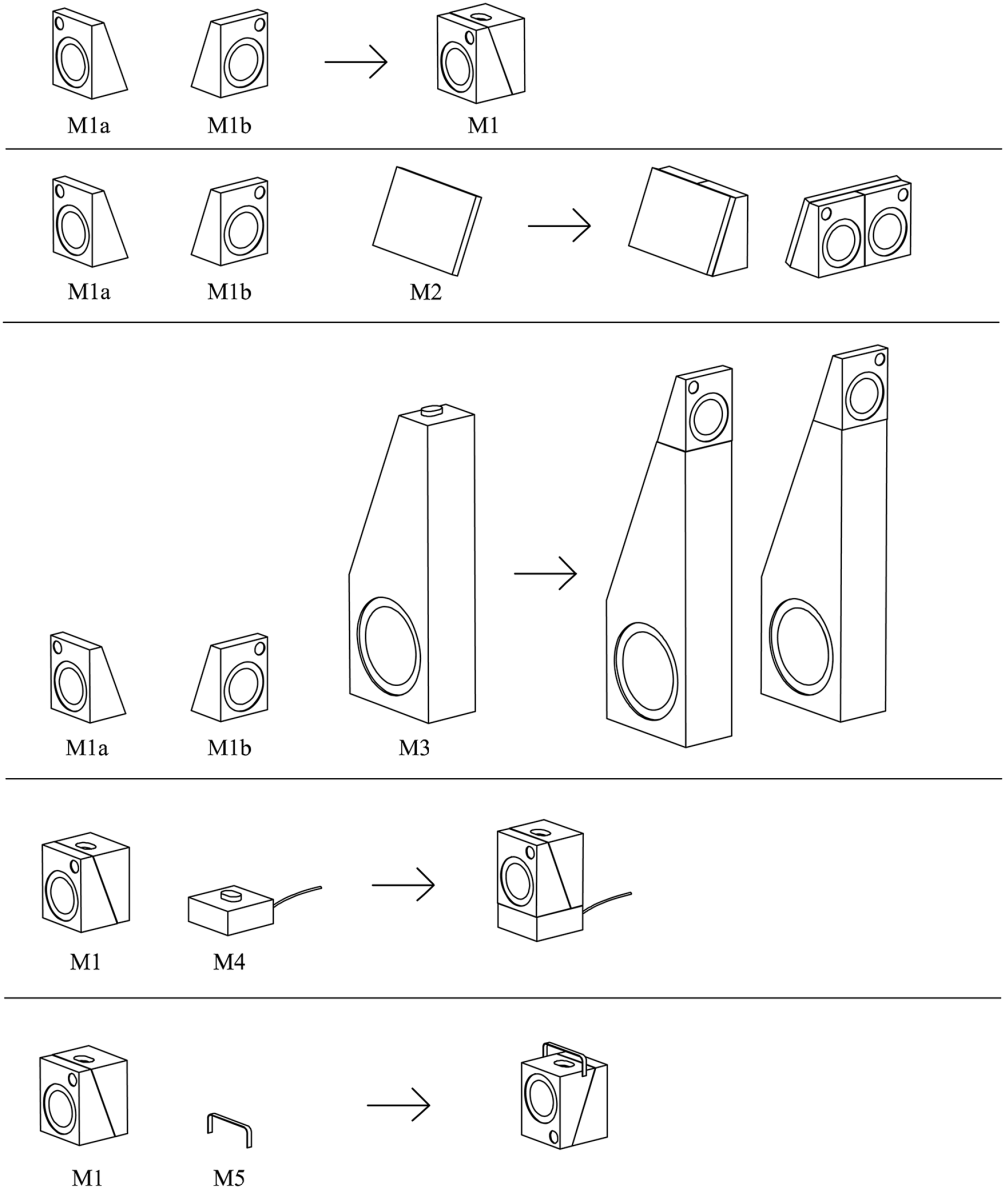
Modularization results and combination diagram.

Based on the modularization results, the conceptual prototype design of the audio product is carried out. Through modeling software, an initial model is created, resulting in a combined form with functional concepts, as shown in Fig 5.

Fig 5 shows a set of modular audio products and their corresponding functional modules. M1a and M1b are the main functional modules, each containing a 1-inch driver and a 3-inch driver, along with built-in batteries and other associated circuit components. M1a and M1b can be combined into module M1 and paired with handle M5 to form a portable outdoor audio system. M2 is a screen capable of displaying dynamic images, and when M1a and M1b are connected side by side and combined with M2, they become an audio system for home entertainment scenarios. M3 is a bass driver with a 6.5-inch driver, bass reflex port, power amplifier, and other components. M1a and M1b are combined with M3 to create a high-fidelity home theatre audio system. When placed on M4, a charging base with a built-in processor and microphone array, it becomes a smart speaker with features such as voice interaction, smart device control, and even the ability to connect to the internet to access AI models, enhancing voice functionality experience.

## Discussion

As the CE market accelerates towards multi-scenario and diverse demands, maintaining a consistent user experience across different contexts has become a core design challenge. Although modular design has garnered attention for its configurability and enhanced user engagement, existing practices (e.g., Google Project Ara, Dyson, Framework) focus primarily on structural aspects [66], lacking theories and methods to systematically partition modules based on “usage scenario differences.” The main contribution of this study lies in proposing a scenario-driven modularization method that originates from user experience and usage scenarios, capable of systematically mapping user usage requirements under different scenarios to modular structures.

By extending the application boundaries of MFD and integrating AHP, QFD, DPM, and MSRM, this study establishes a complete linkage from scenario requirements → product properties → technical solutions → modular structures. The case verification of audio products indicates that this method not only effectively identifies cross-scenario common modules and scenario-specific modules but also provides an actionable analytical path for product platform strategies. The research results demonstrate that a modular method with usage scenarios as the logical thread can enhance product flexibility and reusability, addressing the deficiency where traditional “structure-driven” modularity fails to respond to “experience differences,” thereby enriching the user experience perspective in modularization and platform research.

From a theoretical perspective, this study reveals the critical role of scenario-based requirements in module partitioning, providing a new logical chain for modular design: driving module partitioning by “usage scenarios” rather than “structural modes.” This perspective complements the long-standing limitation of modular theory’s bias towards manufacturing and structural optimization, offering significant implications for product design and development oriented towards experience and flexibility. Furthermore, the scenario-driven modularization framework offers a new pathway for product platform strategies in the multi-scenario era, expanding their application boundaries within the consumer electronics field.

**Fig 6:**
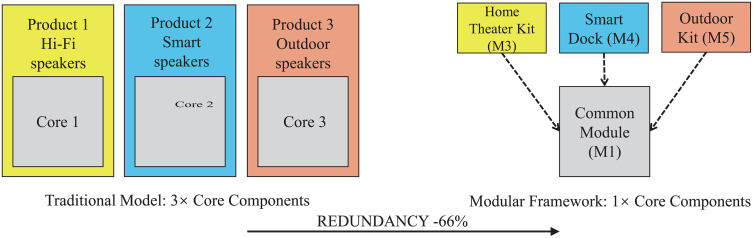
Comparative analysis of component redundancy: Traditional Model vs. Modular Framework. (Left) In the traditional consumption model, satisfying three distinct scenarios (home, smart, outdoor) requires the user to purchase three independent products, leading to the core components (e.g., batteries and PCBs) being purchased three times. (Right) Under the modular framework proposed in this study, by reusing a Common Module (M1) containing the core components, only one set of core components is sufficient to support all scenarios. This theoretically reduces the requirement for high-environmental-impact components by approximately 66%.

From the perspectives of user value and sustainability, this study demonstrates a significant reduction effect on scenario-based modular functional redundancy through a comparative analysis of component redundancy. Under typical usage requirements for three scenarios (home entertainment, smart home, and outdoor portable use), the traditional model necessitates the purchase of three independent products, leading to the repetitive manufacturing and acquisition of core components (e.g., batteries, main control PCBs, wireless communication modules) three times. In contrast, the framework proposed in this study covers all scenario requirements through a single common core module (M1) paired with different functional extension modules (M3, M4, M5), reducing the demand for high-environmental-impact components (e.g., lithium batteries, PCBs) by approximately 66% (as shown in Fig 6). This reduction at the source not only lowers electronic waste generation but also alleviates the economic burden on consumers. Furthermore, the replaceability of modules further enhances product maintainability and extends lifecycles. These results are consistent with existing research conclusions that modularity can reduce material consumption, improve maintainability, and extend product life [6–9,22,23], providing empirical support for the logical link between modularity and sustainable innovation. Future research could conduct more comprehensive Life Cycle Assessments (LCA) or economic evaluations based on this analysis to strengthen the empirical basis of modular design in environmental and cost dimensions.

Although the research results are insightful, several limitations remain. First, user requirements were primarily derived from questionnaire surveys, which may struggle to capture deep-level contextualized needs; future work could combine in-depth interviews and focus groups to enhance insight depth. Second, the MSRM currently relies on expert judgment and has not yet incorporated weighting or sensitivity analysis; future research could further optimize algorithms or employ AI-assisted optimization to address more complex multi-scenario demands. Additionally, this study used audio products as the validation case; its applicability to a broader range of consumer electronics, such as smart wearables and home appliances, requires further verification. Finally, the scenario-driven modularization method proposed herein is primarily intended for the conceptual design phase; module interface standards, CMF design, and production cost trade-offs remain critical issues for practical implementation. Future work could combine industrial practice to explore platform-based interface standards, cross-module compatibility, and life cycle assessment to promote the sustainable application of this framework in the consumer electronics industry.

## Conclusions

From a product design perspective, this study proposes a functional modularization partitioning method centered on usage scenarios and user experience, providing a new complement to traditional structure-centric modular design. This method enables designers to rapidly conduct multi-scenario modular redesign based on a preliminary understanding of the product structure, thereby effectively supporting subsequent conceptual design, functional optimization, and production planning.

Based on the existing MFD theoretical framework, this study integrates AHP, optimizes the QFD and DPM judgment matrices, and further constructs the MSRM, forming a systematic process from user requirement identification to functional module partitioning. Through a questionnaire survey, the study identified four main usage scenarios—daily leisure, home entertainment, smart home, and outdoor activities—and determined users’ key functional requirements, such as ease of operation, multiple sound effect adjustments, and smart integration. Subsequently, AHP analysis was used to determine the weights of each requirement. QFD and DPM were employed to analyze the correlations between technical solutions and product properties. Finally, technical solutions were matched to different scenarios via the MSRM, forming functional module combinations oriented towards multiple scenarios. The audio product case verification demonstrates the method’s effectiveness and good operability within the consumer electronics field.

This study aims to provide flexible modular solutions for diverse usage environments to meet personalized needs. Unlike traditional modular methods for electromechanical products that emphasize structure and component partitioning, this study focuses more on the definition and optimization of functional modules starting from usage scenarios. Case results show that this method can significantly enhance product user experience and flexibility. Furthermore, the research reveals the potential value of scenario-based modularity in sustainability: by providing selectable functional modules, users do not need to purchase multiple devices for different scenarios. Meanwhile, the replaceable module structure helps reduce the overall scrappage rate, lower electronic waste, and extend product lifecycles, thereby reinforcing corporate social responsibility and promoting the formation of sustainable consumption patterns. The scenario-driven modularization framework proposed in this study not only expands the theoretical boundaries of modular design for consumer electronics but also provides a systematic and actionable design path for product development in the multi-scenario era.

## Supporting information

S1 File AHP pairwise comparison matrices. The file contains the geometric mean data derived from the expert judgments.
(XLSX)
